# Polyamines Influence Mouse Sperm Channels Activity

**DOI:** 10.3390/ijms22010441

**Published:** 2021-01-04

**Authors:** Lorena Rodríguez-Páez, Charmina Aguirre-Alvarado, Norma Oviedo, Verónica Alcántara-Farfán, Edgar E. Lara-Ramírez, Guadalupe Elizabeth Jimenez-Gutierrez, Joaquín Cordero-Martínez

**Affiliations:** 1 Laboratorio de Bioquímica Farmacológica, Departamento de Bioquímica, Escuela Nacional de Ciencias Biológicas, Instituto Politécnico Nacional, Ciudad de Mexico 11340, Mexico; lorena_rpaez@yahoo.com.mx (L.R.-P.); charmina_burana@hotmail.com (C.A.-A.); veroalf@yahoo.com (V.A.-F.); elizabeth.jg@hotmail.com (G.E.J.-G.); 2 Unidad de Investigación Médica en Inmunología e Infectología, Centro Médico Nacional, La Raza, IMSS, Ciudad de Mexico 02990, Mexico; naoviedoa@yahoo.com.mx; 3 Unidad de Investigación Biomédica de Zacatecas, Instituto Mexicano del Seguro Social (IMSS), Zacatecas 98000, Mexico; elarar0700@hotmail.com

**Keywords:** polyamines, spermatozoa, soluble adenylate cyclase, channels

## Abstract

Polyamines are ubiquitous polycationic compounds that are highly charged at physiological pH. While passing through the epididymis, sperm lose their capacity to synthesize the polyamines and, upon ejaculation, again come into contact with the polyamines contained in the seminal fluid, unleashing physiological events that improve sperm motility and capacitation. In the present work, we hypothesize about the influence of polyamines, namely, spermine, spermidine, and putrescine, on the activity of sperm channels, evaluating the intracellular concentrations of chloride [Cl^−^]i, calcium [Ca^2+^]i, sodium [Na^+^]i, potassium [K^+^]i, the membrane V_m_, and pHi. The aim of this is to identify the possible regulatory mechanisms mediated by the polyamines on sperm-specific channels under capacitation and non-capacitation conditions. The results showed that the presence of polyamines did not directly influence the activity of calcium and chloride channels. However, the results suggested an interaction of polyamines with sodium and potassium channels, which may contribute to the membrane V_m_ during capacitation. In addition, alkalization of the pHi revealed the possible activation of sperm-specific Na^+^/H^+^ exchangers (NHEs) by the increased levels of cyclic AMP (cAMP), which were produced by soluble adenylate cyclase (sAC) and interact with the polyamines, evidence that is supported by in silico analysis.

## 1. Introduction

Sperm from mammals are produced and differentiate in the testis. At this stage, the sperm cell possesses all of the enzymatic machinery needed to synthesize the polyamines [1]. Polyamines are ubiquitous polycationic compounds; human seminal spermine has a concentration of 3 mM, while spermidine and putrescine have approximately ten times less [2]. These small molecules are highly charged at physiological pH [3] and supposedly enter the cytoplasm through cell-surface heparin sulfate proteoglycans and endocytosis [4]. Once the sperm cells have differentiated, they leave the testis, emerge, and pass through the epididymis. Sperm are morphologically mature at this point, but they lack of fertilization capacity, which is acquired in the epididymis [5,6]. While passing through the epididymis, sperm become fertile, but when this takes place, they lose the capacity to synthesize polyamines, which is attributed to the low and almost undetectable activity of S-adenosylmethionine decarboxylase (EC 4. 1. 1. 50.) and spermidine synthase (EC 2. 1. 5. 16.) [1,7]. Then, the intracellular concentrations of polyamines dramatically decrease [8]. Once the sperm pass through the epididymis, they are stored in the vas deferens waiting to be ejaculated, without any contact with extracellular polyamines [1,6]. Important structures of the male reproductive system include seminal vesicles and the prostate. This gland is connected with the vas deferens [2], and when the process of ejaculation begins, it secretes part of the seminal fluid, which contains water, ions (H^+^, Cl^−^, Na^+^, and HCO_3_^−^) [9], and polyamines. Once the polyamines have entered the cytoplasm, they can interact with a wide variety of molecular targets such as nucleic acids and proteins [3]. Their interactions with different channels of somatic cells include the modulation and blockade of some types of potassium and calcium channels [3,10]. Sperm possess specific channels allowing the flow of ions in response to continuous changes in their environment. This in/efflux of ions allows rapid modification of the intracellular concentrations of ions (calcium, chloride, potassium, and sodium) and membrane potential (V_m_) [11,12]. An in/efflux of ions, changes in V_m_, and alkalization of intracellular pH (pHi) result in the transduction pathways that trigger capacitation [11,13]. During the journey of the spermatozoa from the vas deferens to the female tract, the sperm contact the seminal fluid, which has a different osmolarity from vas deferens [14,15]. In addition, an event critical to capacitation is the increase of intracellular concentration of cyclic AMP (cAMP), which is mainly performed by the soluble adenylate cyclase (sAC); in turn, this is supposedly modulated by an increase of HCO_3_^−^ provided by a sperm-specific Na^+^/H^+^ exchanger (sNHE) [16,17,18]. A possible response of the sperm channels to the change of osmolarity in seminal fluid and the activity of sAC may be modulated by the polyamines. 

In the present work, we hypothesize that polyamines may influence the activity of sAC, which in turn modulates ion homeostasis by the alkalization of pHi. To test this hypothesis, we evaluate the effects of spermine, spermidine, and putrescine on the intracellular concentrations of chloride [Cl^−^]i, calcium [Ca^2+^]i, sodium [Na^+^]i, and potassium [K^+^]i in order to identify regulatory mechanisms mediated by the polyamines on the activity of the sperm ionic channels under capacitation and non-capacitation conditions. Furthermore, we have evaluated the effect of the polyamines on Vm and pHi under capacitation and non-capacitation conditions. Following our hypothesis that polyamines may influence the activity of sAC, we performed an in silico analysis with polyamines and sAC. 

## 2. Results

### 2.1. Sperm Viability Is Not Affected by Low Concentrations of Polyamines

**Figure 1:**
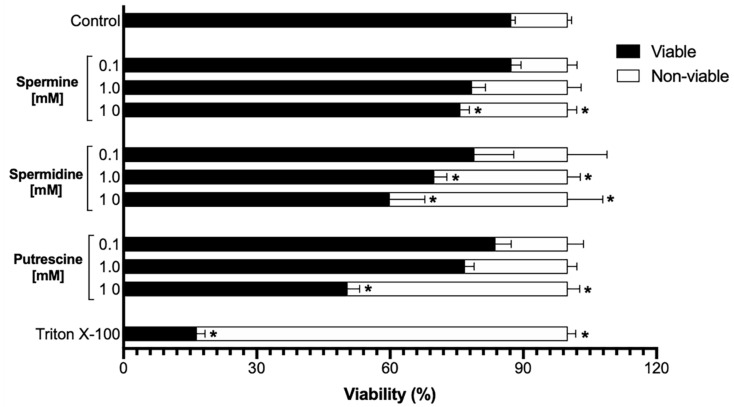
The sperm viability is not affected by low concentrations of polyamines. The graph shows the effect of different concentrations of polyamines on sperm viability. The highest concentrations of polyamines (10 mM) decrease the sperm viability, and this concentration exceeds the physiological concentrations found in seminal fluids. Spermidine affected the sperm viability in a middle concentration (1.0 mM); likewise, this concentration was excluded, along with the highest concentrations to continue with further experiments. A control to induce sperm non-viability was performed using Triton X-100 [0.1%]. Values are the mean ± SEM, *n* = 3. (*) indicates a meaning difference. Data analysis was performed using Tukey’s multiple comparison test, *p* < 0.05.

Sperm suspensions were incubated under capacitation conditions during 90 min in the presence or absence of polyamines. At a low concentration of each polyamine (0.1 mM), sperm viability was not affected (Figure 1). However, at a higher concentration (1.0 mM), viability was affected, with spermidine showing a statistically significant difference compared with the sample control (Figure 1). Finally, at the highest concentration (10 mM), all polyamines showed a statistically significant effect on sperm viability (Figure 1). According to these results, we selected the following concentrations of the polyamines for further experiments: spermine (1.0 mM), spermidine (0.1 mM), and putrescine (1.0 mM). 

### 2.2. Polyamines Decrease the [Cl^−^]i under Capacitation Conditions

**Figure 2:**
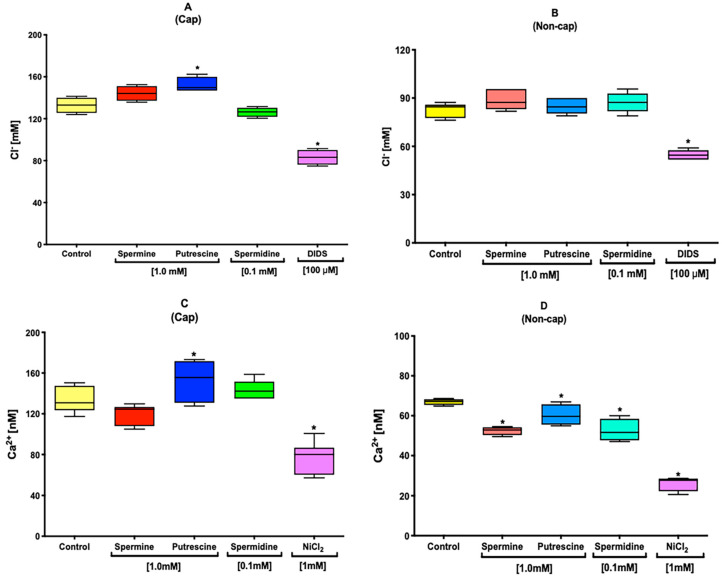
Polyamines decrease the [Cl^−^]i and increase the [Ca^2+^]i under capacitation conditions. The graph shows that putrescine induces an increase of the Cl^−^ influx under capacitation conditions (**A**); meanwhile, the other polyamines did not affect the Cl^−^ influx. However, any of the polyamines induced a change in [Cl^−^]i under non-capacitation conditions (**B**). A control to inhibit general Cl^−^ channels was performed using 100 µM of 4,4-diisothiocyanatostilbene-2,2-disulfonic acid disodium salt hydrate (DIDS). The graph shows that putrescine induced an increase of the Ca^2+^ influx under capacitation conditions (**C**). Nevertheless, under non-capacitation conditions, neither of the polyamines induced an increase of the Ca^2+^ influx (**D**); on the contrary, all polyamines induced a decrease of the Ca^2+^ influx (**D**). A control to inhibit voltage-dependent Ca^2+^ channels was performed using 1 mM NiCl_2_. Values are the mean ± SEM, *n* = 3. (*) indicates a meaning difference. Data analysis was performed using Tukey´s multiple comparison test, *p* < 0.05.

After selecting the optimal polyamine concentration, [Cl^−^]i was evaluated. The results showed that the presence of putrescine induces a significant increase of the Cl^−^ influx under capacitation conditions (Figure 2A). Meanwhile, the other polyamines did not affect the Cl^−^ influx. Nevertheless, none of the polyamines induced a change in [Cl^−^]i under non-capacitation conditions (Figure 2B). As a control, the inhibition of general Cl^−^ channels was performed using 100 µM of 4,4-diisothiocyanatostilbene-2,2-disulfonic acid disodium salt hydrate (DIDS).

### 2.3. Polyamines Increase the [Ca^2+^]i under Capacitation Conditions

The results showed that the presence of putrescine induced an increase of the Ca^2+^ influx under capacitation conditions (Figure 2C). This rise in [Ca^2+^]i was statistically significant when compared with the control sample. However, under non-capacitation conditions, none of the polyamines induced an increase of the Ca^2+^ influx (Figure 2D); instead, they induced a decrease of it (Figure 2D). As a control, the inhibition of voltage-dependent Ca^2+^ channels was performed using 1 mM of NiCl_2_.

### 2.4. Polyamines Increase the [Na^+^]i under Capacitation Conditions

**Figure 3:**
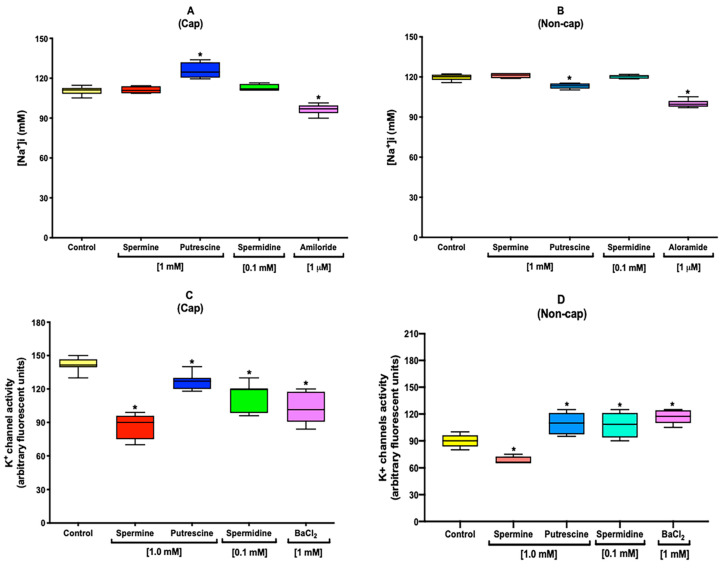
Polyamines increase the [Na^+^]i and decrease the [K^+^]i under capacitation conditions. The graph shows that putrescine induces an increase of the Na+ influx under capacitation conditions (**A**). On the contrary, under non-capacitation conditions, putrescine induced a decrease of [Na^+^]i; meanwhile, the other polyamines did not induce any meaning change (**B**). A control to inhibit general Na^+^ channels was performed using 1 μM of amiloride. The graph shows that the presence of all polyamines induced a decrease of the [K^+^]i under capacitation conditions (**C**) in which spermine was the polymine that induced a marked effect (**C**). Moreover, under non-capacitation conditions, spermine also induced a decrease of the [K^+^]i (D); nevertheless, putrescine and spermidine induced an increase of [K^+^]i (**D**). A control to inhibit general K^+^ channels was performed using 1 mM of BaCl_2_. Values are the mean ± SEM, *n* = 3. (*) indicates a meaning difference. Data analysis was performed using Tukey´s multiple comparison test, *p* < 0.05.

Following the finding that the presence of polyamines did not influence [Cl^−^]i and [Ca^2+^]i under non-capacitation conditions, [Na^+^]i was next evaluated. The results showed that the presence of putrescine induced a significant increase of the Na^+^ influx under capacitation conditions (Figure 3A). In contrast, under non-capacitation conditions, putrescine induced a significant decrease of [Na^+^]i, while the other polyamines did not induce any meaning change (Figure 3B). As a control, the inhibition of general Na^+^ channels was performed using 1 μM of amiloride.

### 2.5. Polyamines Decrease the [K^+^]i under Capacitation Conditions

Under non-capacitation conditions, polyamines influenced the Na^+^ flux. Next, we proceeded to evaluate the [K^+^]i. The results showed that the presence of all polyamines induced a decrease of the [K^+^]i under capacitation conditions (Figure 3C), with spermine being the polyamine inducing a marked effect (Figure 3C). The reduction in [K^+^]i was statistically significant when compared with control sample. Moreover, under non-capacitation conditions, spermine also induced a decrease of the [K^+^]i (Figure 3D). Putrescine and spermidine induced an increase of [K^+^]i, which was statistically significant when compared with the control sample (Figure 3D). As a control, the inhibition of general K^+^ channels was performed using 1 mM of BaCl_2_.

### 2.6. Polyamines Induce Changes in the V_m_ under Capacitation and Non-Capacitation Conditions

**Figure 4:**
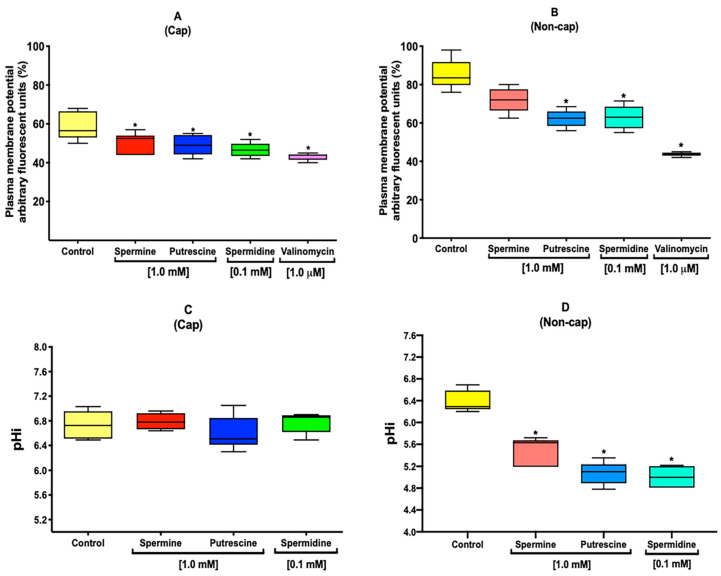
Polyamines induce changes in the V_m_ and influence the alkalization of pHi under capacitation and non-capacitation conditions. The presence of spermine, putrescine, and spermidine induce hyperpolarization of the V_m_ with a meaning difference compared with control (**A**). Moreover, under non-capacitation conditions, putrescine and spermidine induce hyperpolarization of the V_m_ with a meaning difference compared with the control (**B**). The graph shows no meaning difference between the samples incubated in the presence of all polyamines compared with the control (**C**) under capacitation conditions. Nevertheless, under non-capacitation conditions, all polyamines induced an acidification of the pHi (**D**). Values are the mean ± SEM, *n* = 3. (*) indicates a meaning difference. Data analysis was performed using Tukey’s multiple comparison test, *p* < 0.05.

We observed changes in Na^+^ and K^+^ flux, which could influence the membrane V_m_. Under capacitation conditions, the results showed that the presence of all polyamines induced a hyperpolarization of V_m_, which was significantly different from the control (Figure 4A). Under non-capacitation conditions, putrescine and spermidine induced significant hyperpolarization of the V_m_ (Figure 4B). A control to hyperpolarize the V_m_ was performed using 1 μM of valinomycin.

### 2.7. Polyamines Influence the Alkalization of pHi during Capacitation

After observing changes in V_m_, pHi was evaluated. The results showed no significant difference between the samples incubated in the presence of all polyamines compared with the control (Figure 4C) under capacitation conditions. However, under non-capacitation conditions, all polyamines induced an acidification of the pHi (Figure 4D).

### 2.8. Polyamines Decrease the ATP Level

**Figure 5:**
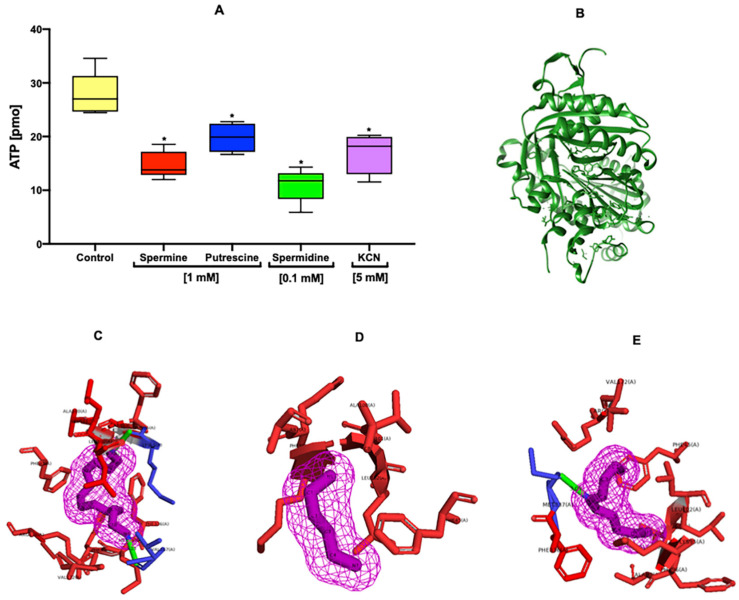
Polyamines decrease the ATP level due the possible interaction with sperm soluble adenylate cyclase (sAC). The graph shows a marked decrease of ATP level in the sperm incubated in the presence of spermine and spermidine (**A**). Nevertheless, the presence of putrescine did not induce a meaningful decrease of ATP level (**A**). The KCN^−^ (5 mM) was used as a negative control to uncouple the respiratory chain. The docking for spermine, putrescine, and spermidine with sAC (**B**) showed binding energies of −4.4, −3.3, and −4.0 kcal/mol respectively, which could be due to the linear chemical structure that makes them very flexible (**C**–**E**). The best binding poses for the three polyamines are mainly thorough hydrophobic interactions shown in red (**C**–**E**). Among them, the best compound was spermine with −4.4 kcal/mol, which showed two hydrophilic interactions (shown in green) with Val167 and Lys95 amino acids, and it could explain the obtained binding energy. Values are the mean ±SEM, *n* = 3. (*) indicates a meaning difference. Data analysis was performed using Tukey´s multiple comparison test, *p* < 0.05.

The results showed a marked decrease of ATP level in the sperm incubated in the presence of spermine and spermidine (Figure 5A). However, the presence of putrescine did not induce a significant decrease of ATP level (Figure 5A). KCN^−^ [5 mM] was used as a negative control to block the respiratory chain.

### 2.9. In Silico Interaction of Polyamines with Sperm sAC

The first docking between the sAC (Figure 5B) and bithionol ligand clearly reproduced the experimental binding mode of on the bicarbonate binding site with an RMSD of 1.18 Å. The predicted binding energy was of −7.5 kcal/mol. The subsequent docking of spermine, putrescine, and spermidine showed weak binding energies compared with that of bithionol (−4.4, −3.3, and −4.0 kcal/mol), which could be due to the chemical structure that makes them very flexible (Figure 5C–E). The best binding poses for the three polyamines are mainly through hydrophobic interactions (Figure 5C–E). Among them, the best compound was the spermine with −4.4 kcal/mol, which showed two hydrophilic interactions with the carbonyl of the Val167 and Lys95 amino acids, potentially explaining the obtained binding energy.

## 3. Discussion

In human, seminal spermine has a concentration of 3 mM; meanwhile, spermidine and putrescine has approximately ten times less [2]. These concentrations must be essential for sperm function and lack any toxic effects [19,20]. In somatic cells, the toxicity of polyamines has not been well elucidated, because these cells express the polyamine oxidase (EC 1.5.3.11). Polyamine oxidase catalyzes the conversion from spermine and spermidine to acrolein, which is a toxic compound [21]. This makes it difficult to evaluate the toxicity of polyamines themselves. On the other hand, somatic cells also express cationic amino acid transporters (SLC3A2), which impede the intracellular accumulation of high concentrations of polyamines [22]. Moreover, human spermatozoa exhibit very low expression of spermidine synthase (EC 2.5.1.16) and S-adenosyl-L-methionine decarboxylase [7]. Other key enzymes in polyamine metabolism are only present in seminal fluid [1,2,7]. Therefore, to evaluate the toxicity of the polyamines in vitro, we assayed them at a range of concentrations (0.1, 1.0, and 10 mM) and found that only the highest concentrations of polyamines (10 mM) decreased the sperm viability (Figure 1). These high assay concentrations exceed the physiological concentrations found in seminal fluid [2], so they could be toxic to sperm. Nevertheless, spermidine at 1.0 mM affected the viability of sperm (Figure 1). As such, this concentration was excluded, along with the highest concentrations upon continuing with further experiments.

Chloride channels play a major role in regulating sperm motility, capacitation, and acrosomal reaction [12,23]. The homeostasis of Cl^−^ during capacitation is regulated by different structural families of channels. Among these, we can describe the cystic fibrosis transmembrane conductance regulator (CFTR) channels, which are present in mouse sperm [12,24]. In addition, CFTR dysfunction is related to the loss of fertility and cystic fibrosis [25]. During capacitation, [Cl^−^]i increases [12], and the results showed an increase of [Cl^−^]i induced by the putrescine (Figure 2A). Spermine and spermidine did not induce any change under capacitation conditions (Figure 2A). However, under non-capacitation conditions, none of the polyamines was able to increase [Cl^−^]i (Figure 2B), which could be attributable to the NaHCO_3_ present in the capacitation media or via the possible interaction of polyamines with casein kinase II (CK2), which also modulates the activity of CFTR. CFTR is composed of five domains: two membrane-spanning domains (MSDs), two nucleotide-binding domains (NBDs), and a regulatory (R) domain. While the MSDs form the channel pore, phosphorylation of the R domain determines channel activity, and ATP hydrolysis by the NBDs controls the channel-gating properties [12,24]. In addition, the activity of CFTR is mediated by phosphorylation carried out by CK2, which is present in mouse sperm [26]. CK2 is a ubiquitous, pleiotropic, and constitutively active Ser/Thr protein kinase with polyamine-binding subunits [27]. 

Ca^2+^ is an ion that is an important ion for sperm physiology. Under capacitation conditions, the sperm undergo a rise of the intracellular concentration of this cation [28,29], which is mediated by the activation of different channels such as transient receptor potential canonical (TRPC) [30]. Among the members of the structural TRPC family, we found that TRPC2, which is present in mouse sperm, actively contributes to the rise of [Ca^2+^]i that leads to the acrosomal reaction [30]. Sperm incubated in the presence of putrescine under capacitation conditions showed a significant increase of [Ca^2+^]i (Figure 2C) compared with the control. However, under non-capacitation conditions, we observed a decrease of [Ca^2+^]i in the presence of all polyamines (Figure 2D), which was possibly also attributable to the lack of NaHCO_3_ in non-capacitation medium and not directly to the presence of polyamines. 

Na^+^ channels are important to sperm physiology because they lower the permeability to Na^+^ and may contribute to the membrane hyperpolarization during the capacitation of mouse sperm. In addition, during capacitation, the [Na^+^]i decreases, which may be attributable to the epithelial Na^+^ channels (ENaC) present in mouse sperm [23,31]. Under capacitation conditions, we observed an increase of [Na^+^]i only in the presence of putrescine (Figure 3A). However, under non-capacitation conditions, we observed a significant decrease of [Na^+^]i in the samples incubated with the same polyamine (Figure 3B). [Cl^−^]i and [Ca^2+^]i, showed a significant increases in the presence of NaHCO_3_ under capacitation conditions. These conditions could also be relevant to the rise of [Na^+^]i, since ENaC could be modulated by the activation of CFTR, which is also modulated by the rise of cAMP promoted by the presence of NaHCO_3_ [32].

Some K^+^ channels open in response to the presence of [Ca^2+^]i or other intracellular signaling molecules. Slo3 is a sperm-specific K^+^ channel found in mouse sperm, which is modulated by intracellular pH, but it is also voltage-dependent, helping to restore V_m_ prior to capacitation [12,33]. During capacitation, the mouse sperm membrane suffers a hyperpolarization influenced by the [K^+^]e; meanwhile, there is an alkalization of pHi and efflux of K^+^ is performed by Slo3, suggesting a decrease of [K^+^]i [12,34]. The results showed that all polyamines induced a reduction of [K^+^]i (Figure 3C); moreover, under non-capacitation conditions, we also observed a decrease of [K^+^]i (Figure 3D), suggesting an increase in the K+ permeability produced by the presence of spermine. Recently, it was reported that exogenous spermidine could block the MthK potassium channel in bacteria [35], which would explain the possible blockade of Slo3 and the reduction of [K^+^]i by polyamines.

In mouse sperm, under capacitation conditions, the sperm membrane potential hyperpolarizes, which could be influenced by the activation of Slo3, followed by a decrease of the [K^+^]i [12,36], a loss of permeability of Na^+^, and a decrease of [Na^+^]i [31,37]. These results suggest hyperpolarization in the presence of all polyamines (Figure 4A). In addition, under no-capacitation conditions, polyamines (putrescine and spermidine) induced a hyperpolarization of the sperm membrane (Figure 4B). It has already been reported that drugs such as amiloride, genistein, and valinomycin induce membrane hyperpolarization under non-capacitation conditions [38], and polyamines could induce the same effect. This evidence is supported by the results presented in Figure 3, where it can be observed that changes in [Na^+^]i and [K^+^]i flux may contribute to the membrane V_m_, which also influences the modulation of pHi during capacitation [36,39]. The presence of polyamines allows the alkalization of pHi (Figure 4C) under capacitation conditions; nevertheless, under no-capacitation conditions, none of the polyamines induce an increase of pHi (Figure 4D), which is possibly attributable to the absence of NaHCO_3_ in non-capacitation medium. The maintenance of pHi is essential for cell functions, and changes in this parameter could trigger important physiological events; likewise, the regulation of sperm pHi is performed by membrane H^+^/ HCO_3_^−^ transporters [9,40]. H^+^ carriers include transporters of the solute carrier 9 (SLC9) family, which are highly effective at regulating pHi. These cation exchangers constitute a family of membrane proteins commonly named Na^+^/H^+^ exchangers (NHEs) [9,41]. In mouse sperm, the pHi appears to be regulated by NHEs, and the activity of these transporters is modulated by cAMP [18,39]. There are two classes of adenylyl cyclases in mammalian cells: G protein-regulated transmembrane adenylyl cyclases (tmACs) and Ca^2+^-HCO_3_^−^-regulated soluble adenylyl cyclases (sACs) [39,42]. On the other hand, previous studies provide evidence in which polyamines may modulate the activity of sAC. Specifically, the addition of spermine and spermidine increased the levels of cAMP in human sperm, suggesting their possible modulation of sAC activity [43,44,45]. This evidence is supported by docking for spermine, putrescine, and spermidine, which suggests an interaction of the polyamines with the sAC (Figure 5B–E). This could be supported by previous studies in which polyamines improved sperm motility and capacitation [1,46,47]. Moreover, the decrease of ATP level in the presence of polyamines (Figure 5A) suggests that an important proportion of ATP has been used to synthesize cAMP, in addition to that used for the acquisition of capacitation and hypermotility [17]. 

**Figure 6:**
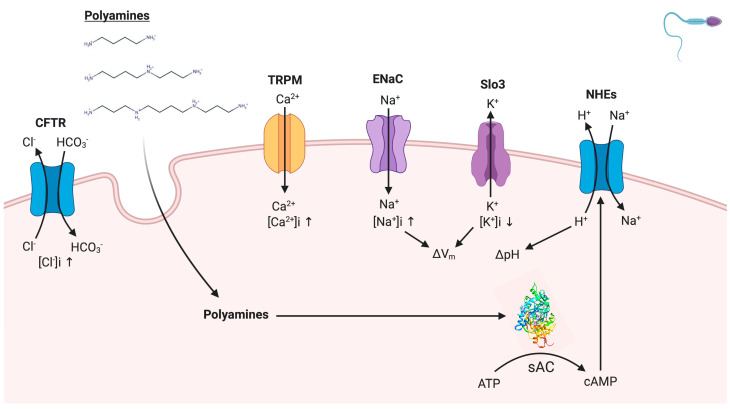
Possible modulation of sAC by polyamines. Polyamines (spermine, spermidine, and putrescine) enter into the cytoplasm by endocytosis and did not influence directly the activity of calcium and chloride channels. However, the results suggest an interaction between polyamines with sodium and potassium channels, which may contribute to the establishment of membrane V_m_ during capacitation. In addition, the alkalization of pHi reveals the possible activation of Na^+^/H^+^ exchangers (NHEs) by the involvement of sAC, which potentially interact with the polyamines.

In conclusion (Figure 6), the increases of [Ca^2+^]i and [Cl^−^]i appear to be independent of the presence of polyamines during capacitation. However, the results of this work suggest the interaction of polyamines with sodium and potassium channels, which may contribute to the membrane V_m_ during capacitation. In addition, the alkalization of pHi suggests a possible activation of NHEs by the increased levels of cAMP produced by sAC, which potentially interacts with the polyamines. Finally, as a perspective of this work, we will attempt to relate the activity of sAC with the variation of polymines levels in seminal fluid, which is possibly found in infertile men and patients with diabetes.

## 4. Materials and Methods 

### 4.1. Materials and Reagents

All reagents were purchased from Sigma Chemical Co. (St. Louis, MO, USA) or Mallinckrodt Baker (Phillipsburg, NJ, USA), except for DiBAC_4_(3), which was purchased from Biotium Inc. (Fremont, CA, USA). Fura-2 AM, CoroNa^TM^ Green, MQAE, BCECF-AM, and the FluxOR potassium ion channel assay were purchased from Thermo Fisher, Scientific (Waltham, MA, USA).

### 4.2. Animal Procedures

Adult male mice (CD1 albino) were obtained from the ENCB-IPN Vivarium, weighing from 20 to 25 g. They were maintained in polypropylene cages at room temperature, with a normal light/dark cycle, and provided standard commercial food and water ad libitum. The investigation and animal protocols were approved by The Institutional Ethics Committee of Escuela Nacional de Ciencias Biológicas of the Instituto Politécnico Nacional (protocol no. 902144 in Januaty the 7th of 2014) in accordance with the Guide for the Care and Use of Laboratory Animals, published by the National Institutes of Health (Bethesda, MD, USA).

### 4.3. Sperm Preparation

Using 154 mM of NaCl, sperm were obtained by puncture at 37 °C. Immediately thereafter, the sperm were centrifuged and resuspended in modified Tyrode´s medium at a pH 7.6 (120 mM NaCl, 2.8 mM KCl, 11.9 mM NaHCO_3_, 0.36 mM NaH_2_PO_4_, 0.49 mM MgCl_2_, 0.25 mM sodium pyruvate, 20 mM sodium lactate) 1.8 mM CaCl_2_, 5.56 μM glucose, and 1 mg/mL albumin to achieve capacitation conditions (capacitation medium). The same medium was modified by replacing 11.9 mM NaHCO_3_ with 25 mM HEPES and removing glucose and albumin to achieve non-capacitation conditions (non-capacitation medium). The final sperm concentration for each experiment was 8–10 × 10^6^ spermatozoa/mL. Experiments were performed in triplicate at 37 °C [48].

### 4.4. Evaluation of Sperm Viability

Sperm suspensions were incubated in capacitation Tyrode’s medium at pH 7.6 and 37 °C for 90 min in the presence or absence of different concentrations of spermine, spermidine, and putrescine (0.1, 1.0, and 10 mM). Once the incubation was complete, to evaluate the viability and membrane integrity, double staining using fluorescein diacetate (FDA) and propidium iodide (PI) was performed on the sperm suspension. The possible toxic effects of the polyamines on the treated sperm were measured by flow cytometry using FACSAria cell sorter (Becton Dickinson, Towson, MD, USA) equipped with a 488 nm laser beam. The results were analyzed by FACSDiva software (Becton Dickinson). To confirm the effect of polyamines on sperm viability, 0.1% Triton X-100 was used as a negative control. According to the results, one concentration of each polyamine was selected for further experiments [49].

### 4.5. Intracellular Cl^−^ Concentration Determination in Sperm Population

Sperm suspensions were loaded for 30 min with 10 mM Cl^−^ fluorescent indicator MQAE at 37 °C. After incubation, these suspensions were washed by centrifugation (6000× *g* for 6 min) to discard the MQAE excess. Thereafter, the sperm suspensions were resuspended with fresh capacitation or non-capacitation Tyrode medium. Immediately thereafter, the sperm suspensions were transferred into a 96-well plate, and the polyamines were added at the selected concentrations: spermine at 1.0 mM, spermidine at 0.1 mM, and putrescine at 1.0 mM. After 90 min of incubation under capacitation or non-capacitation conditions, fluorescence was measured using a Synergy 2 Multi-Function Microplate Reader (Bio-Tek Instruments, Winooski, VT, USA) with excitation at 350 nm and emission at 450 nm. As a control, the inhibition of general Cl^−^ channels was performed using 100 µM of 4,4-diisothiocyanatostilbene-2,2-disulfonic acid disodium salt hydrate (DIDS). All obtained data were interpolated in an activity calibration curve, which was constructed using the same sperm concentration (8–10 × 10^6^ spermatozoa/mL) and incubated in capacitation or non-capacitation Tyrode´s medium, containing different concentrations of Cl^−^ (0, 20, 40, 60, 80, and 100 mM) [37,50].

### 4.6. Intracellular Ca^2+^ Concentration Determination in Sperm Population

Sperm suspensions were loaded simultaneously for 30 min with 1 μM of Fura-2 AM and 0.05% of pluronic acid F-127. Once the incubation had been completed, the sperm suspensions were washed by centrifugation (6000× *g* for 6 min) to remove the excess of Fura-2 AM and pluronic acid F-127 and resuspended with fresh capacitation or non-capacitation Tyrode´s medium. Immediately thereafter, the sperm suspensions were transferred into a 96-well plate, and polyamines were added at the selected concentrations. After 90 min of incubation under capacitation or non-capacitation conditions, fluorescence was measured using a Synergy 2 Multi-Function Microplate Reader (Bio-Tek Instruments, Winooski, VT, USA) at an alternating wavelength of 340–380 nm and an emission wavelength of 510 nm. As a control, the inhibition of voltage-dependent Ca^2+^ channels was performed using 1 mM NiCl_2_. All obtained data were interpolated in an activity calibration curve, which was constructed using the same sperm concentration (8–10 × 10^6^ spermatozoa/mL) and incubated in capacitation or non-capacitation Tyrode´s medium, containing different concentrations of Ca^2+^ (0, 50, 100, 200, 400, 800, and 1000 nM) [51].

### 4.7. Intracellular Na^+^ Concentration Determination in Sperm Population

Sperm suspensions were loaded for 30 min with 10 μM of CoroNa^TM^ Green. Once the incubation had been completed, the sperm suspensions were washed by centrifugation (6000× *g* for 6 min) to remove the excess of CoroNa^TM^ Green and resuspended with fresh capacitation or non-capacitation Tyrode´s medium. Immediately thereafter, the sperm suspensions were transferred into a 96-well plate, and polyamines were added at the selected concentrations. After 90 min of incubation under capacitation or non-capacitation conditions, fluorescence was measured using a Synergy 2 Multi-Function Microplate Reader (Bio-Tek Instruments, Winooski, VT, USA) with excitation at 492 nm and emission at 516 nm. As a control, the inhibition of general Na+ channels was performed using 1 μM of amiloride [23]. All obtained data were interpolated in an activity calibration curve that was constructed using the same sperm concentration (8–10 × 10^6^ spermatozoa/mL) and incubated in capacitation or non-capacitation Tyrode´s medium, containing different concentrations of Na^+^ (0, 15, 30, 50, 100, and 150 mM) [52].

### 4.8. Intracellular K^+^ Concentration Determination in Sperm Population

Experiments were performed with FluxOR Potassium Ion Channel Assay, in accordance with the manufacturer´s protocol. Briefly, sperm suspensions were incubated for 60 min with loading buffer at 37 °C protected from the light. Immediately thereafter, the sperm suspensions were washed by centrifugation (6000× *g* for 6 min), and the loading buffer was replaced with capacitation or non-capacitation Tyrode´s medium. Polyamines were added to them at the selected concentrations to continue with the incubation during 90 min at 37 °C protected from the light. Thereafter, Tyrode´s medium was removed from the sperm suspension and immediately replaced with assay buffer. Immediately thereafter, sperm suspensions were transferred into a 96-well plate, and fluorescence was measured using a Synergy 2 Multi-Function Microplate Reader (Bio-Tek Instruments, Winooski, VT, USA) with excitation at 490 nm of excitation and emission at 525 nm. As a control, the inhibition of general K^+^ channels was performed using 1 mM of BaCl_2_ [53].

### 4.9. Evaluation of Sperm V_m_ in Sperm Population

Sperm suspensions were loaded for 60 min with the fluorescent indicator DiBAC_4_(3) at 37 °C. Once the incubation had been completed, the suspensions were washed by centrifugation (6000× *g* for 6 min) to remove the excess DiBAC_4_(3) and resuspended with fresh capacitation or non-capacitation Tyrode´s medium. Immediately thereafter, the sperm suspensions were transferred into a 96-well plate, and the polyamines were added at the selected concentrations. After 90 min of incubation under capacitation or non-capacitation conditions, fluorescence was measured using a Synergy 2 Multi-Function Microplate Reader (Bio-Tek Instruments, Winooski, VT, USA) with excitation at 492 nm of excitation and emission at 516 nm. A control to hyperpolarize the V_m_ was performed using 1 μM of valinomycin [38,54].

### 4.10. Quantification of Sperm pHi in Sperm Population

Sperm suspensions were loaded for 30 min with the fluorescent indicator of pHi, BCECF-AM, at 37 °C. Once the incubation had been completed, the sperm suspensions were washed by centrifugation (6000× *g* for 6 min) to remove the excess of BCECF-AM. In individual experiments, cells were and resuspended with fresh capacitation, non-capacitation, and modified Tyrode´s medium. Immediately thereafter, the sperm suspensions were transferred into a 96-well plate, and polyamines were added at the selected concentrations. After 90 min of incubation under capacitation or non-capacitation conditions, fluorescence was measured using a Synergy 2 Multi-Function Microplate Reader (Bio-Tek Instruments, Winooski, VT, USA) at an excitation wavelength of 490 nm and an emission wavelength of 535 nm. A pH calibration curve was made with the same sperm concentrations (8–10 × 10^6^ spermatozoa/mL) to interpolate all data previously obtained. Sperm suspensions were incubated in capacitation or non-capacitation Tyrode´s medium with adjusted pH (7.0, 7.2, 7.4, 7.6, 7.8, and 8.0). Before recording, the sperm suspensions were permeabilized using 0.1% Triton X-100 [54].

### 4.11. Quantification of Sperm ATP Level

Incubation was performed for 90 min under capacitation or no-capacitation conditions, with and without spermine at 1.0 mM, spermidine at 0.1 mM, and putrescine at 1.0 mM. Immediately thereafter, the sperm suspensions (25 μL) were transferred to a conical tube, and the extraction buffer (0.1 M Tris-HCl, 4 mM EDTA, pH 7.8) was added, followed by boiling at 100 °C for 5 min. Subsequently, the sperm suspensions were centrifuged at 20,000× *g* for 5 min, and the supernatant was recovered. Using a luciferase bioluminescence assay, the ATP levels were measured in accordance with the manufacturer´s protocol (ATP Bioluminescent Somatic Cell Assay Kit FL-ASC; Sigma Chemical Co.). Luminescence was measured using a Synergy 2 Multi-Function Microplate Reader (Bio-Tek Instruments, Winooski, VT, USA) [55].

### 4.12. In Silico Study of the Interaction between Polyamines and sAC

The crystal structure of human sAC was obtained from the Protein Data Bank (PDBID: 5D0R). This molecule was co-crystallized with the allosteric inhibitor bithionol [2,2′-sulfonylbis(4,6-dichlorophenol)] located at the bicarbonate binding site [56]. The 5D0R pdb file was prepared as a receptor, removing the ligand and other molecules with the software USCF Chimera [57]. Polar hydrogens and Gasteiger charges were added with the AutoDockTools to proteins and ligands [58]. The same process was performed for the ligands spermine, spermidine, putrescine, as well as the bithionol. First docking was performed with the Autodock Vina software [59] to obtain the search space coordinates using the biothionol as a query. The search space was determined at 1 angstrom (Å) spacing, with the center coordinates located as follows: x = 15.927, y = 25.754, z = 0.016, and box size of X,Y,Z of 10. These coordinates were used to dock the three ligands tested. The binding energy for the first pose was analyzed in comparison with the ligand bithionol with the help of LigPlot-Pymol v.4.5.3 software [60].

### 4.13. Statistical Analysis

Statistical analysis was performed using GraphPad Prism 8 (GraphPad Software). All data are shown as the mean±SEM. Statistical significance of the results was determined using one-way ANOVA followed by Dunnett´s test. Differences were considered significant at *p* < 0.05.

## Data Availability

The data presented in this study are available on request from the corresponding author. The data are not publicly available due to privacy.

## Abbreviations

sAC	soluble adenylate cyclase
[Cl^−^]i	intracellular concentrations of chloride
[Ca^2+^]i	intracellular concentrations of calcium
[K^+^]i	intracellular concentrations of potassium
[Na^+^]i	intracellular concentrations of sodium
DIDS	4,4′diisothiocyanatostilbene-2,2′-disulfonic acid disodium salt hydrate
V_m_	membrane potential
cAMP	cyclic adenosine mono phosphate
